# Case report: Severe hepatic fibrosis induced by chronic cholestasis of congenital biliary dilation treated by laparoscopic surgery after immunonutrition support– An infantile case

**DOI:** 10.3389/fped.2022.1101000

**Published:** 2023-01-12

**Authors:** Koshiro Sugita, Shun Onishi, Mitsuru Muto, Nanako Nishida, Ayaka Nagano, Masakazu Murakami, Toshio Harumatsu, Koji Yamada, Waka Yamada, Takafumi Kawano, Satoshi Ieiri

**Affiliations:** Department of Pediatric Surgery, Research Field in Medical and Health Sciences, Medical and Dental Area, Research and Education Assembly, Kagoshima University, Kagoshima, Japan

**Keywords:** chronic hepatitis, compensated liver cirrhosis, congenital biliary dilation, infant, laparoscopic hepaticojejunostomy

## Abstract

**Introduction:**

In some patients with congenital biliary dilation (CBD), biliary cirrhosis has been reported to rapidly progress from the neonatal period to the infantile period. We herein report an infantile case of CBD that showed severe biliary cirrhosis at the diagnosis, which was treated by laparoscopic surgery.

**Case presentation:**

A 16-month-old girl underwent conservative therapy for liver dysfunction and cholangitis on a remote island of our prefecture. She was transferred to our hospital after the detection of a huge dilated common bile duct on imaging at the previous hospital. Contrast-enhanced computed tomography showed a dilated common bile duct (maximum diameter: 5 cm), thus suggesting CBD. However, her laboratory data on admission showed a poor nutritional status and severe liver dysfunction (Alb, 2.5 mg/dl; AST, 79 IU/L; ALT, 43 IU/L; γ-GTP, 491 mg/dl; D-bil, 0.3 mg/dl; CHE, 90 IU/L; NH_3_, 123 μg/dl). We initially performed laparoscopic exploration and bile drainage *via* the gallbladder, noting severe hepatic fibrosis resembling end-stage liver cirrhosis. After placing a drainage tube in the gallbladder, cholangiography was performed. Cholangiography showed Todani type IVa CBD with pancreaticobiliary maljunction. Contrast agent flowing into the duodenum could not be confirmed. The patient received liver-supporting therapy and nutritional support for 7 weeks before definitive surgery. Following the improvement of the hepatic synthetic capacity (Alb, 4.0 mg/dl; AST, 82 IU/L; ALT, 78 IU/L; γ-GTP, 157 mg/dl; D-bil, 0.2 mg/dl; CHE, 232 IU/L; NH_3_, 75 μg/dl), we performed extrahepatic bile duct excision and hepaticojejunostomy laparoscopically. Laparoscopic surgery was successfully performed along with liver biopsy. Histopathologically, the liver specimen showed chronic hepatitis and fibrosis (F3A2). Biliary scintigraphy showed good bile excretion at postoperative day 15. The postoperative course uneventful, and the patient was discharged on the 23rd day after surgery. A needle liver biopsy six months later showed mild improvement of chronic hepatitis and fibrosis (F2-3A1). The patient was regularly followed at the outpatient clinic.

**Conclusions:**

Severe liver fibrosis was suspected to be continuous cholestasis of CBD after birth. CBD with severe liver fibrosis may avoid liver transplantation by two-stage surgery with hepatoprotection therapy and immunonutritional support.

## Introduction

Congenital biliary dilatation (CBD) is more in some Asian region as than in the Western countries, including United States, and some Asian populations have an incidence as high as 1 in 1,000 births (1–5). In contrast, some Asian populations have an incidence as high as 1 in 1,000 births. There is a marked sex difference, with the rate in females being 3–4 times higher than that in males (6–8). Todani et al. classified the extrahepatic bile duct into four types and the intrahepatic bile duct into one type (9). Type V is treated synonymously with Caroli disease, one of the so-called fibropolycystic diseases, but differs from congenital hepatic fibrosis in the thickness of the bile ducts that are damaged (10, 11). Almost 80% of choledochal cysts (CCs) are now discovered prenatally or in infancy (12), but it has been reported that biliary cirrhosis rapidly progresses from the neonatal period to the infantile period in some cases of CBD (13). We herein report an infantile case of CBD that showed severe biliary cirrhosis at the diagnosis, which was treated by laparoscopic surgery.

## Case presentation

### Preoperative course

The patient was a 16-month-old girl. No prenatal abnormalities were noted. She visited a nearby doctor on a remote island for fever and was treated with antibiotic therapy as an outpatient. She gradually developed clouding of consciousness and dyspnea. She was transferred to our hospital on the 13th day after the onset of symptoms due to a huge dilated common bile duct that was detected on imaging at the previous hospital.

### First treatment approach

**Figure 1:**
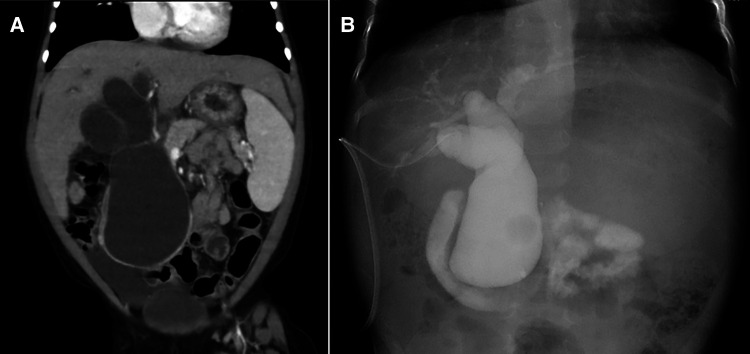
Diagnostic imaging findings (**A**) contrast-enhanced computed tomography (**B**) cholangiography.

Contrast-enhanced computed tomography showed dilation of the common bile duct (maximum diameter: 5 cm) (Figure 1A), suggesting CBD. However, her laboratory data on admission showed severe liver disfunction (AST, 79 IU/L; ALT, 43 IU/L; γ-GTP, 491 mg/dl; D-bil, 0.3 mg/dl; Alb 2.5 mg/dl; CHE, 90 IU/L; NH_3_, 123 μg/dl) (Table 1). The Child-Pugh classification was equivalent to Grade A–B when combined with the fact that she had consciousness disturbance, a history of vitamin K treatment, and her laboratory findings at the time of admission. We initially performed laparoscopic exploration and bile drainage *via* the gallbladder, noting severe hepatic fibrosis resembling end-stage liver cirrhosis (Supplementary File S1). A 5-mm 30° laparoscope was inserted through an umbilical incision along with a 5 mm trocar with a multichannel port device (E.Z Access/LAP-PROTECTOR minimini; Hakko Co., Ltd., Tokyo, Japan). Since the view of the lower liver space could not be obtained, a 3-mm port was additionally inserted through the EZ Access to secure the view. The liver had many hoop-like notches on both lobes, which is a finding of liver sclerosis. A 3-mm port was additionally inserted into the right upper abdomen and the gallbladder was pulled out from the port wound. A double purse suture was applied with 4–0 PDS outside the body and an incision was made, and then an 8-Fr balloon catheter was inserted, and the tip was placed in the common bile duct. After placing a drainage tube in the gallbladder, cholangiography was performed. We confirmed continuity between dilation of the intrahepatic bile ducts and the common bile duct, and it consistent with findings of biliary dilatation. There was no gallbladder atrophy, which is seen in I cyst-type biliary atresia (Figure 1B). Cholangiography revealed Todani type IVa CBD with pancreaticobiliary maljunction. Then, the patient received liver-supporting therapy and nutritional support for 7 weeks before definitive surgery.

**Table 1:** Changes in blood test data.

	Admission	5 days after drainage	10 days after drainage	1 month after drainage	5 days after Lap-HJ	10 days after Lap-HJ	Before discharge	6 months later After Lap-HJ
AST (IU/L)	79	70	54	82	98	73	50	46
ALT (IU/L)	43	28	24	78	171	86	45	29
γGTP (mg/dl)	491	262	256	157	111	78	79	31
D-bil (mg/dl)	0.3	0.2	0.2	0.2	0.1	0.1	0.1	0.03
CHE (IU/L)	90	133	145	232	205	160	255	357
Alb (mg/dl)	2.5	2.8	3.2	4	3.3	3.2	4.1	4.1
Pt (%)	104	81	79	68	54	74	98	82
NH_3_ (μg/dl)	123	123	124	75	-	122	116	76

PT, contains modification with vitamin K.

### Secondary treatment approach

**Figure 2:**
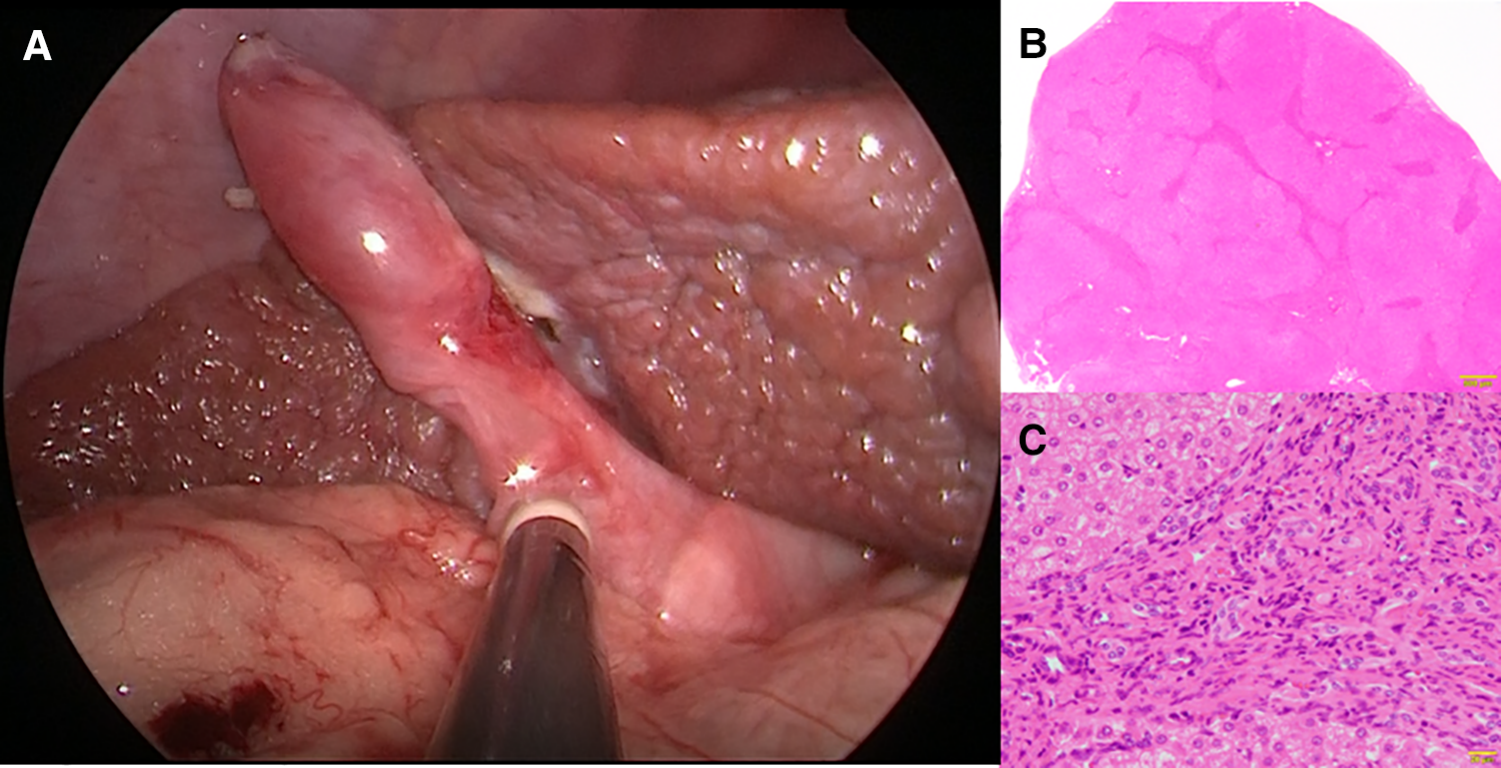
Histopathological findings at hepaticojejunostomy. Chronic hepatitis and fibrosis, F3, A2. (**A**) Intraoperative findings (**B**) An enlarged portal area and bridging fibrosis were observed (Hematoxylin Eosin staining ×10). (**C**) Proliferation of regenerated bile ducts and the infiltration of neutrophils and lymphocytes were observed (Hematoxylin Eosin staining ×200).

Following the improvement of the hepatic synthetic capacity (Alb, 4.0 mg/dl; AST, 82 IU/L; ALT, 78 IU/L; γ-GTP, 157 mg/dl; D-bil, 0.2 mg/dl; CHE, 232 IU/L; NH_3_, 75 μg/dl) (Table 1), we performed extrahepatic bile duct excision and hepaticojejunostomy laparoscopically (Supplementary File S2). Laparoscopic choledochal cyst excision was performed using five ports. Under general anesthesia, the patient was placed in the broad base position, and the operator stand to the right side of the patient. A 10-mm 30° laparoscope was inserted through an umbilical incision along with a trocar with a multichannel port device (E.Z Access/LAP-PROTECTOR minimini; Hakko Co., Ltd., Tokyo, Japan). Pneumoperitoneum was established with 8-mm Hg CO_2_ insufflation. Three additional trocars and a 2.4-mm needle-type grasper (Teleflex, Morrisville, NC, USA) were inserted into the right upper abdomen (operator's left hand, 3.5 mm) and at the right side of the umbilicus (operator's right hand, 5 mm), the left lateral abdomen (assistant's left hand, 3.5 mm), and the left upper abdomen (assistant's right hand, 2.4 mm). The dilated CBD was dissected and then taping was performed. After imaging the lower bile duct and confirming that the bile duct on the side of the pancreas was sufficiently detached, the lower bile duct was clipped and transected. Subsequently, after dissection and transection of the cystic duct, the hepatic duct just above the dilated common hepatic duct was transected. The jejunum was then extracted from the umbilical wound, and Roux-en Y jejunojejunostomy was performed. The mucosa and serosa of the opened hole was approximated using 6–0 absorbable sutures to secure hepaticojejunostomy. The jejunum was pulled up through the retro-colic route. Both the posterior and anterior walls were approximated using interrupted intracorporeal knot-tying with 5–0 absorbable sutures. Laparoscopic surgery was successfully performed along with liver biopsy. Histopathologically, the liver specimen showed chronic hepatitis and fibrosis (F3A2) based on the new Inuyama classification (14) (Figures 2A,B). F4 is defined as liver cirrhosis, but it presents clinical findings as disturbed consciousness with hyperammonemia and intraoperative findings as advanced liver fibrosis, and we clinically diagnosed to be almost equivalent to liver cirrhosis in the compensation stage. Biliary scintigraphy showed good bile excretion on postoperative day 15 (Supplementary File S3). The postoperative course was uneventful and the patient was discharged on the 23rd day after surgery.

### Clinical course after discharge

**Figure 3:**
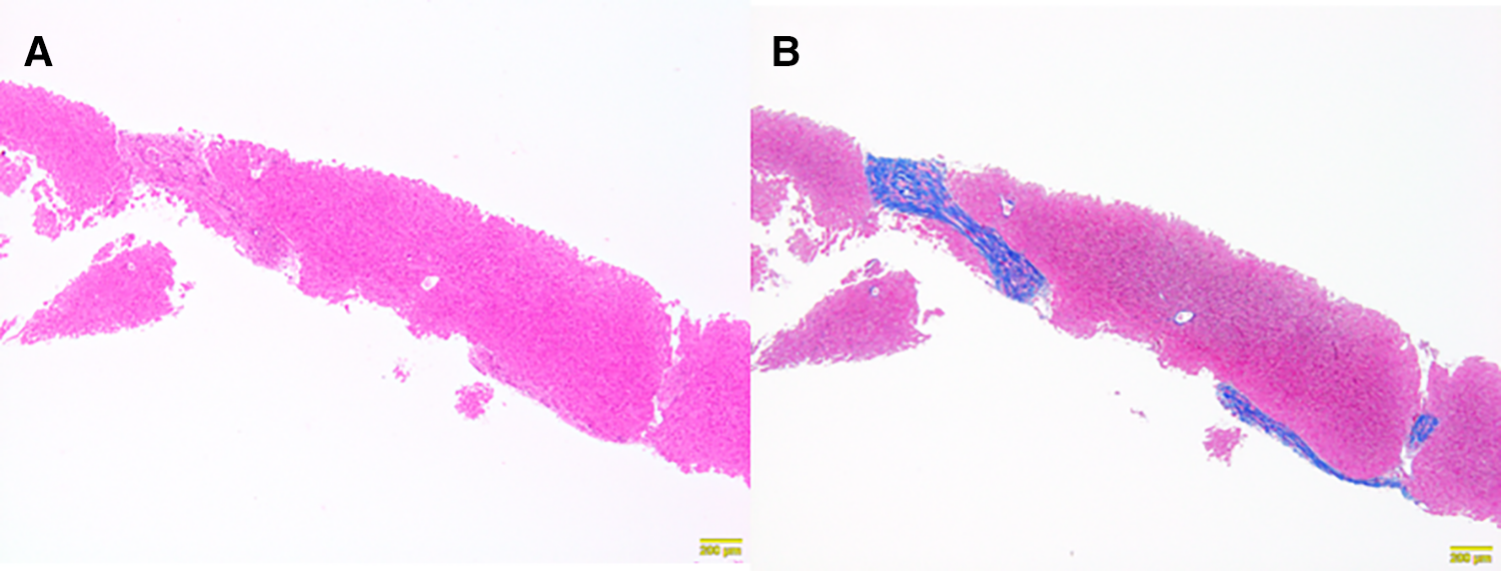
Histopathological findings of a needle liver biopsy specimen obtained six months later. Chronic hepatitis and fibrosis, F2–3, A1. (**A**) Persistent portal area enlargement was observed. The inflammatory findings had improved (Hematoxylin Eosin staining ×10). (**B**) Persistent bridging fibrosis was observed (AZAN staining ×10).

At 6 months after laparoscopic extrahepatic bile duct resection and hepaticojejunostomy, she was readmitted and underwent needle liver biopsy to confirm the morphological improvement after surgery. After discharge from the hospital, her hepatic function normalized, and her cholinesterase level, which was low before surgery, showed a tendency toward improvement; however, her NH_3_ level remained above the normal range (Table 1). A histopathological examination showed mild improvement of chronic hepatitis and fibrosis (F2-3A1) (Figures 3A,B). The patient was regularly followed at the outpatient clinic.

## Discussions

The primary goal of CBD treatment is of resection the extrahepatic bile duct and reconstruction of the bile flow route to prevent the potential occurrence of malignant tumors of the bile duct. However, the possibility of liver fibrosis progressing—leading to end-stage cirrhosis requiring liver transplantation—cannot be ruled out. In Todani type IV, such as our case, the CBD shows intrahepatic bile duct dilatation. CBD is mainly represented by Todani types I and IV. The surgical treatment for CBD is resection of the dilated bile duct and biliary reconstruction. Biliary reconstruction can be performed by means of Roux-en-Y hepaticojejunostomy, hepaticoduodenostomy, or jejunal interposition heptaticoduodenostomy (5). In the case of type I, there is a possibility of malignancy, the incidence of which different differs between Asian and Western countries. However, due to the lack of long-term patient information, the subset with a low risk of developing malignant tumors is unknown. Thus, resection of the extrahepatic bile duct seems to be recommended worldwide as a basic treatment strategy, as it is for type IVa (3).

Liver fibrosis in patients with CBD has been shown to worsen with age, and there are reports recommending surgery in early infancy (15). Obstructive bile ducts with impaired duodenal outflow, such as in our case, may progress rapidly from liver fibrosis to cirrhosis. Suita et al. reported that obstructive cholangiopathy is the main pathology in the neonatal period, causing acute and chronic cholangitis due to reflux of pancreatic juice. It is speculated that exacerbation of cholestatis due to this inflammation is the underlying cause of liver fibrosis (16). Type IVa CBD is likely to be associated with the progression of liver fibrosis, and prompt surgical intervention is recommended, especially for neonatal and infantile cases with type IVa because irreversible liver cirrhosis can occur, even in the infantile period (17). We think that the process leading to hepatic fibrosis is due to the degree of cholestasis. Our case was a type IV CC with no prenatal diagnosis and was not diagnosed until it became symptomatic, and it took a long time to be diagnosed. The cholestasis, in our case, may be mild in the early postnatal period. Reflux of pancreatic juice causes inflammation and exacerbates cholestasis, and the liver began with mild inflammation, and after a prolonged course, chronic inflammation accumulated and caused liver fibrosis. It is possible that the interrelationship with the varying with or without congenital cyst, cholestasis, and refluxed pancreatic secretions into the bile may have contributed to the differences in the course of the disease. There is no need to rush the timing of surgery, as long as it is possible to prevent malignancy; however, in symptomatic cases it is necessary to assess the condition of the liver and decide a therapeutic strategy.

Our search of the PubMed database using the keywords [congenital biliary dilation, liver transplantation], yielded 46 reports. Most of the reports describing cases in which CBD led to liver transplantation involved patients with Caroli disease, which is a rare inherited disorder characterized by cystic dilatation of the bile ducts within the liver. On the other hand, type I–IVa CBD was reported in only 6 patients by Hori et al. (13) and 1 patient by Wang et al. (18). The classifications of these 7 cases included type Ia (*n* = 1), type Ic (*n* = 2) and type IVa (*n* = 4), and their ages ranged from 0.7 to 37.3 years. The grade of liver fibrosis ranges from F2 to F4. The causes leading to liver transplantation were postoperative portal vein thrombosis, postoperative anastomotic stenosis, repeated shunt surgery, recurrent cholangitis and pancreatitis, and slowly progressive postoperative cirrhosis. Many of these causes underwent liver transplantation due to the worsening clinical course after extrahepatic bile duct resection and hepaticoenterostomy although these CC patients are not indicated for liver transplantation at the time of initial surgery. We evaluate that our case was not indication for liver transplantation at the time of diagnosis. Liver transplantation may be avoided with appropriate therapeutic intervention in many of the liver fibrosis patients with CC. However, there are various reports of liver cirrhosis in the neonatal period and reports of the progression of postoperative liver fibrosis (13); thus, careful postoperative follow-up is required. This patient's condition at the time of admission suggested liver fibrosis, and there was concern about the progression of the disease in the perioperative period; thus, hepatoprotective therapy with minimally invasive drainage was performed first. In our case, hepatic deviating enzymes were improved by blood test, but fibrosis of F2–3 remained histologically, and ammonia was not completely improved. This may suggest that normalization of hepatic deviant enzymes does not necessarily indicate complete recovery of liver function. Ammonia was thought to have a stronger correlation with histological evaluation than hepatic enzymes, and it was thought that ammonia should be measured during follow-up. Ishimaru et al. reported a case with postoperative liver cirrhosis that improved with standard surgical strategies (19). Some CBD patients with liver cirrhosis at surgery may also be able to avoid surgery with standard surgical treatment, but malnutrition—represented by hypoalbuminemia and a decreased liver function—may affect the progression of postoperative liver cirrhosis and outcomes in postoperative period as well as the perioperative period (20). Oral nutrition combined with branched-chain amino acids was given for liver cirrhosis. Recovery of liver function and improvement of nutritional status associated with appropriate amino acid supplementation may have had a positive impact on postoperative uneventful outcomes. Immnonutrition is useful for improving postoperative outcomes in adult surgical diseases (21), but it seems that appropriate perioperative nutritional management and determination of surgical timing are also important in the pediatric field. Laparoscopic cholecystostomy followed by laparoscopic cyst excision is a useful and safe procedure for the treatment of CBD patients whose symptoms do not improve (22). A hepatoprotective treatment strategy, such as two-stage surgery for CBD patients with liver cirrhosis—as in our case—may also help reduce postoperative complications and improve postoperative outcomes in comparison to initial radical surgery.

## Data Availability

The original contributions presented in the study are included in the article/**Supplementary Material**, further inquiries can be directed to the corresponding author/s.
