# Supplementary figures and images for: Case report: Severe hepatic fibrosis induced by chronic cholestasis of congenital biliary dilation treated by laparoscopic surgery after immunonutrition support– An infantile case
Source: Front Pediatr. 2023 Jan 12;10:1101000. doi: 10.3389/fped.2022.1101000 (PMC9878591; doi:10.3389/fped.2022.1101000)

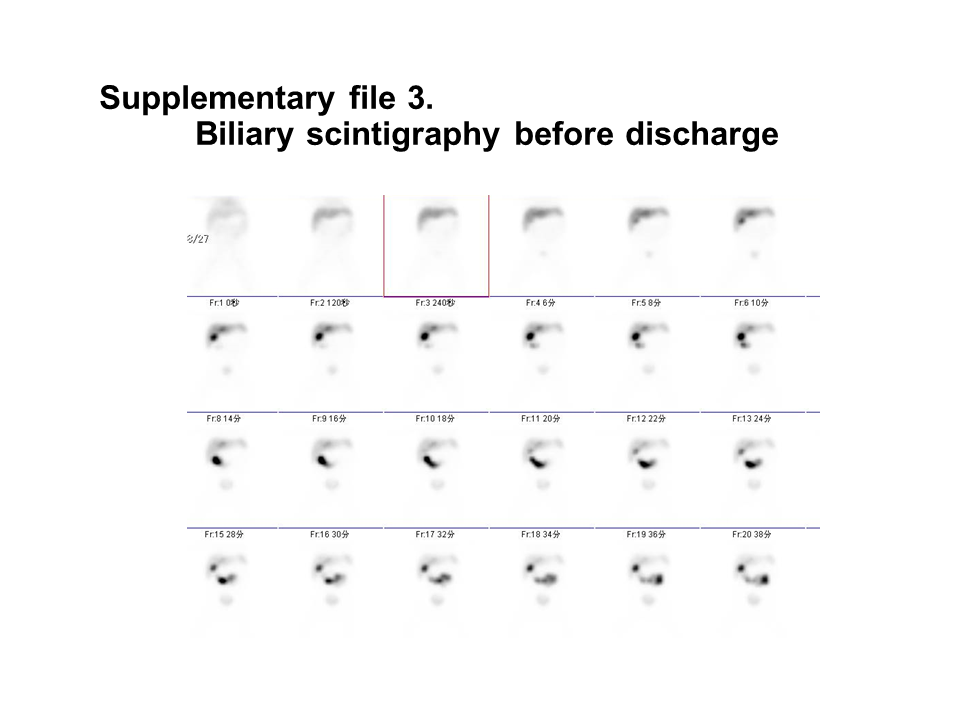

Supplement: Supplementary file 3 [file Image1.tif]
